# Tumor-derived extracellular vesicle nucleic acids as promising diagnostic biomarkers for prostate cancer

**DOI:** 10.3389/fonc.2023.1201554

**Published:** 2023-06-29

**Authors:** Yanjun Diao, Bingbing Zhu, Ting Ding, Rui Li, Jinjie Li, Liu Yang, Lei Zhou, Xiaoke Hao, Jiayun Liu

**Affiliations:** Department of Clinical Laboratory Medicine, Xijing Hospital, Fourth Military Medical University, Xi’an, Shaanxi, China

**Keywords:** prostate cancer, extracellular vesicles, nucleic acids, diagnosis, biomarker

## Abstract

Liquid biopsy as a non-invasive method has a bright future in cancer diagnosis. Tumor-related extracellular vesicles (EVs) and their components (nucleic acids, proteins, and lipids) in biofluids may exert multiple functions in tumor growth, metastasis, immune escape, and angiogenesis. Among all the components, nucleic acids have attracted the most interest due to their simplicity of extraction and detection. In this review, the biological functions of EVs in prostate cancer (PCa) genesis and progression were summarized. Moreover, the diagnostic value of EV RNA markers found in clinical body fluid samples was reviewed, including their trends, challenging isolation methods, and diagnostic efficacy. Lastly, because relatively much progress has been made in PCa, studies on EV DNA markers are also discussed.

## 1 Introduction

As a new and minimally invasive method, liquid biopsy is aimed at isolating genetic material from biological fluids for tumor diagnosis. It is promising to overcome the invasive sampling and heterogeneity problems of tissue biopsy to achieve better screening and monitoring of tumor recurrence risk. As one of the three targets of liquid biopsy, these lipid-bilayered vesicles with cellular origins are released into the extracellular region by a variety of cells, including cancer cells. Extracellular vesicles (EVs) can be categorized into exosomes, microvesicles, and apoptotic bodies according to their size, origin, morphology, and mode of release. Tumor-derived EVs are thought to mediate intracellular communication as “horizontal” transfer cargo and reflect their cell-type origin, suggesting they could offer a good source of novel tumor diagnosis biomarkers.

EVs are exciting in a vast range of biofluids (blood, urine, bronchoalveolar lavage fluid, ascites and pleural effusion, breast milk, saliva, etc.) and contain a myriad of functional biomolecules (proteins, lipids, RNA, DNA). Considering EVs in biological fluids generally reflect their parent cells’ information, their unique composition could assist in cancer detection. Compared to lipids and proteins, the easy-to-detect nature of nucleic acids makes them an attractive source for clinical application (1). RNA, in particular, has been the most exploited because it is protected from nucleases by lipid bilayers and is easier to detect using PCR and next-generation sequencing (NGS). Another component of nucleic acids, DNA, has been less studied but has gained more attention in recent years.

Prostate cancer (PCa) has the highest incidence of male genitourinary malignancy and is the most common cancer in Western men. It is well known that the routine screening method prostate-specific antigen (PSA) has many limitations due to its poor specificity. For example, in addition to the elevation in PCa, PSA also increases during anal digital examination, catheter insertion, prostate inflammation, and hypertrophy (2). Therefore, novel, noninvasive, and highly specific diagnostic biomarkers are highly needed. Although it is controversial whether the number of EVs in tumor patients is increased relative to healthy controls, the reported PCa studies are relatively consistent in suggesting that the secretion of EVs in PCa patients is five to 10 times higher than that in healthy subjects or patients with benign prostatic hyperplasia (BPH) (3–5). As an androgen-dependent tumor, androgen deprivation therapy (ADT) is the main standard treatment for PCa and the basis for the combination of various treatment options. Studies have shown that after ADT, EVs isolated from the urine of PCa patients or cell culture supernatant were approximately two- to threefold decreased (6). In terms of quantity, all this evidence suggests the potential of EVs for diagnosis and treatment efficacy prediction for PCa. Therefore, this study took PCa as the object to review the diagnostic value of EV RNA markers found based on clinical body fluid samples, as well as the relevant progress of PCa EV DNA markers in recent years.

## 2 The biological function of EVs in PCa

**Figure 1:**
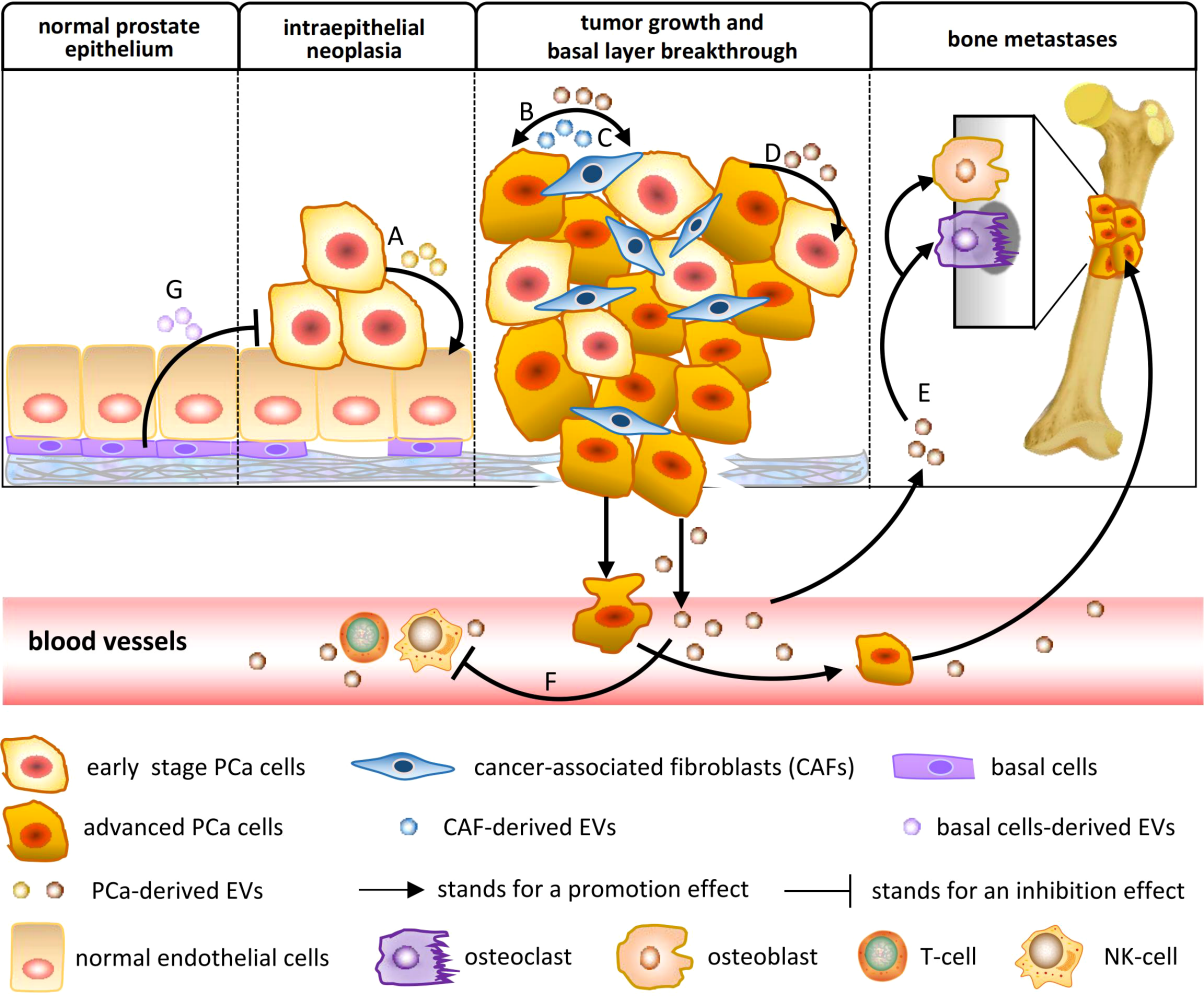
The biological function of EVs in PCa occurrence and development. **(A)** PCa-derived EVs mediate epithelial–mesenchymal transition (EMT), increasing the migration and invasion of noncancerous prostate epithelial cells. **(B)** PCa-derived EVs triggered fibroblast differentiation to a distinctive myofibroblast phenotype, resembling stromal cells isolated from PCa tissue, which support angiogenesis *in vitro* and accelerate tumor growth *in vivo*. **(C)** Cancer-associated fibroblast (CAF)-derived EVs can reprogram the metabolic machinery of PCa, supplying metabolomics for PCa cells and promoting tumor growth. **(D)** PCa-derived EVs confer prosurvival signals to alter the phenotype of other PCa cells in their surrounding environment, including reducing apoptosis, increasing proliferation, inducing migration, and transferring resistance. **(E)** PCa-derived EVs regulate the bone microenvironment by affecting osteoblast and osteoclast differentiation, which facilitates PCa cell proliferation and invasion. **(F)** PCa-derived EVs promote immune suppression and tumor escape through the downregulation of the cytotoxic response of cellular immunity. **(G)** Adipose-derived stromal cell (APS) is known as one of the mesenchymal stem cells (MSCs). APS-derived EVs could inhibit metastatic PCa progression.

The potential of EVs for clinical application may be determined by their biological function. The education function of tumor-related EVs to target cells is through the horizontal transfer of carried biomolecules, which is involved in the modulation of cancer metabolism and the microenvironment. Several studies have confirmed that intercellular transfer of EV cargo changes the phenotype of recipient cells and contributes to PCa occurrence and development. A model of the action mechanism is shown in **Figure 1**, and the literature related to EV function is summarized in **Table 1**.

**Table 1:** Summary of mechanism studies investigating EVs’ function on recipient cells.

Category	EV-generating cells	Biomolecule expression on EVs	Recipient cells	EV function	Reference
PCa-derived EV-mediated EMT	PCa cells lines: LNCaP and PC3	ITGA3 ↑	Noncancerous prostate epithelial cells (prEC)	Inhibition of exosomal ITGA3 reduced the migration and invasion of noncancerous prEC almost completely.	Bijnsdorp et al. (7)
PCa-derived EV-triggered fibroblast differentiation	PCa cells	TGF-β1	Fibroblasts	Prostate cancer exosomes triggered TGFβ1-dependent fibroblast differentiation to a distinctive myofibroblast phenotype resembling stromal cells isolated from cancerous prostate tissue; supporting angiogenesis *in vitro* and accelerating tumor growth *in vivo*.	Webber et al. (8)
CAF-derived EVs could reprogram PCa metabolic machinery	CAFs	Metabolomics including amino acids, lipids, and tricarboxylic acid cycle intermediates	Cancer cells (PCa, pancreatic cancer)	Cancer cells avidly utilized metabolomics delivered by CAF EVs for central carbon metabolism and promoting tumor growth under nutrient deprivation or nutrient-stressed conditions.	Zhao et al. (9)
	Urine samples of 3 PCa patients and 2 healthy men	Not applicable	Prostate CAFs: primary cultures PCF-54 and PCF-55 from 2 PCa tissues	EVs from PCa patients elicited transcriptional changes in CAFs related to cell division regulation, chromosome segregation, and chemokine expression, which showed functional heterogeneity.	Sadovska et al. (10)
PCa-derived EVs could confer prosurvival signals to alter the phenotype of other PCa cells	PCa cells lines: DU145	Not applicable	PCa cells lines: LNCaP	PCa-derived exosomes significantly reduce apoptosis, increase cancer cell proliferation, and induce cell migration in LNCaP and RWPE-1 cells.	Hosseini-Beheshti et al. (11)
	PCa cells lines: DU145 and 22Rv1 docetaxel-resistant variants (DU145RD and 22Rv1RD)	MDR-1/P-gp	PCa cells lines: DU145, 22Rv1, and LNCaP	EVs derived from DU145 and 22Rv1 docetaxel-resistant variants (DU145RD and 22Rv1RD) conferred docetaxel resistance to DU145, 22Rv1, and LNCaP cells, which may be partly due to exosomal MDR-1/P-gp transfer.	Corcoran et al. (12)
PCa-derived EVs could regulate the bone microenvironment by affecting osteoblast and osteoclast differentiation to facilitate bone metastasis	PCa cell	NEAT1 (lncRNA)	Human bone marrow-derived mesenchymal stem cells (hBMSCs)	PCa cell-derived EVs facilitated the activity of alkaline phosphatase (ALP) and mineralization of extracellular matrix and continuously upregulated the levels of RUNX2, ALP, alpha-1 type 1 collagen, and osteocalcin by regulating RUNX2 to induce the osteogenic differentiation of hBMSCs.	Mo et al. (13)
	PCa cell lines: 22Rv1, DU145, and PC-3	NORAD (lncRNA)	Bone marrow stromal cells	NORAD might serve as a competing endogenous RNA (ceRNA) of miR-541-3p to promote PKM2 expression, thereby promoting internalization and transfer of PCa EVs to bone marrow stromal cells, enhancing the development of bone metastasis in PCa.	Hu et al. (14)
	PCa cell lines: DU145	AY927529 ↑ (lncRNA)	Bone marrow stromal cell line (ST2)	Exosome-mediated lncAY927529 could positively regulate CXCL14 levels in ST2 cells and promote PCa cell proliferation and invasion by regulating bone microenvironment.	Li et al. (15)
	PCa cell lines: PC3 and DU145	v-ets erythroblastosis virus E26 oncogene homolog 1	Osteoblasts	PCa-EVs enhanced osteoblast differentiation mainly through the delivery of PCa cell-derived v-ets erythroblastosis virus E26 oncogene homolog 1, which is an osteoblast differentiation-related-transcriptional factor.	Itoh et al. (16)
	PCa cell lines: MDAPCa 2b, C4-2, PC3, and LNCaP AI+F	miR-92a-1-5p	Osteoclasts	miR-92a-1-5p delivered by PCa EVs could downregulate type I collagen expression by directly targeting COL1A1 and thus promoting osteoclast differentiation and inhibiting osteoblastogenesis.	Yu et al. (17)
PCa-derived EVs promote immune escape through downregulating cytotoxic response	PCa cell lines: 22Rv1, PC-3, and LNCaP	Not applicable	Natural killer (NK) and CD8+ T cells	PCa-derived EVs could downregulate NKG2D-mediated cytotoxic response, which promotes immune suppression and tumor escape.	Lundholm et al. (18)
APS-derived EVs could inhibit metastatic PCa progression	Adipose-derived stromal cell (ASC)	miR-145	Metastatic PCa cells	ASC-derived EVs could inhibit PC3M-luc2 cell proliferation and induce apoptosis.	Takahara et al. (19)

The ↑ means that the biomolecule expression was increased on EVs.

At present, a few studies have suggested that the nucleic acid component of EVs could be useful for developing novel biomarkers. In one such study, Hessvik et al. evaluated EVs derived from the cell culture medium of PCa epithelial cell lines PC-3 and the human prostate (noncancer) epithelial cell line RWPE-1. Microarray identified 26 unique miRNAs in the EVs of RWPE-1, 20 unique miRNAs in the EVs of PC-3, and 362 overlapping miRNAs between the two (20). These unique EV miRNAs from noncancer and PCa cell lines could lead to the discovery of novel biomarkers. Furthermore, lots of comparison studies were conducted between different PCa clinical conditions such as aggressiveness [Gleason Score (GS)], metastasis, androgen resistance, and response to antitumor drugs (3, 21). These results demonstrated that EV RNA expression patterns can be related to specific PCa clinical situations, fueling the hope to find more valuable biomarkers to distinguish tumor subtypes and predict treatment response. The other EV nucleic acid component, DNA, was first observed in 2014; it was found later and got less attention than EV RNA (22). In addition, using PCa as a model, it has been proven that in different subtypes of EVs, DNA components are mainly encapsulated in large EVs (1~10 μm diameter) (23). In this review, we systematically summarize studies investigating EV nucleic acids (RNA and DNA) in biofluids of prostate cancer patients and discuss the utility of the identified biomarkers in various clinical scenarios. Furthermore, we outline the biological questions and technical challenges that have arisen from these studies.

## 3 EV RNA biomarker in PCa

At present, due to the simplicity of isolation and detection, nucleic acids have attracted the most interest in EV content. Under such circumstances, research on tumor miRNA marker screening has gradually turned from free-circulating miRNAs to EV-encapsulated miRNAs in body fluids. Compared to free circulating RNA in body fluids, EV RNA has advantages such as: (i) EV double-layer lipid membrane could protect RNA against nuclease degradation (24), which allows for higher level and easier detection; (ii) EVs selectively encapsulate RNA. Matthew et al. have demonstrated the selective sorting of miRNAs into EVs (25). In PCa, Hessvik et al. identified 31 miRNAs only detected in EVs through a comparison of the miRNA profile of EVs vs. parent cells using a PC-3 cell line (20). (iii) For urological tumor diagnosis, urine supplies a noninvasive approach to detect tumor-derived biomarkers. Among the known forms of miRNA packaging, EV-enclosed miRNA is thought to overcome urinary impurity interference and reflect the vital activity of cancer cells. A urine sample is easier to get than blood, has a larger volume and less damage, and may truly be “noninvasive”. In addition, prior prostate massage has been demonstrated to enhance the efficiency of urinary EV detection (26–28).

Based on deep sequencing analysis of the EV RNA subtypes, pioneering work has identified mature miRNA as having the highest proportion, ranging from 29% to 44% of the total EV RNA, with long noncoding RNA (lncRNA) and mRNA accounting for most of the remaining EV RNA (29–31). For RNA marker screening, comparisons have most commonly been made between different cell lines (for example, androgen-sensitive vs. androgen-independent; less aggressive vs. aggressive, etc.) or between different clinical conditions (health control or BPH vs. PCa patients, localized PCa vs. metastatic PCa, castration-sensitive vs. castration-resistant PCa, nonrecurrent PCa vs. recurrent PCa, etc.). Quite all studies demonstrate that such specific cultures or clinical situations can be related to specific expression patterns, and EV RNA biomarker specificity increases with the development of tumors. Therefore, the diagnostic potential of EV RNA markers has received extensive attention. Many studies used a comparison between clinical sample groups to screen EV RNA markers with significant expression differences. However, the high heterogeneity among single papers was relevant to sample type, EV separation method, and specimen size, which concluded that only a few RNAs, such as miR-141, miR-375, or PCA3, had a common trend and most were different. In order to gain the whole picture of the reported PCa RNA biomarkers, we summarized the published screening studies to show the biomarker types, expression trends, experimental design, and conclusions, as shown in **Table 2**. It should be noted that the samples we used here for comparison were all derived from patients’ blood or urine and did not contain cell culture supernatant, although there was also a study comparing EV miRNA profiles between PCa cell lines (PC-3) and noncancerous lines (RWPE-1) cultured *in vitro* (20).

**Table 2:** Summary of clinical studies investigating EV nucleic acid targets as potential PCa biomarkers.

Targets evaluated	Body fluid sources	Number of participants	EV isolation methods	EV target testing methods	Diagnostic value/outcome	Reference	
miR-141 ↑	Serum	20 PCa vs. 20 BPH vs. 20 HC A follow-up cohort: 51 PCa (20 mPCa vs. 30 LPCa)	ExoQuick Exosome Precipitation Solution (System Biosciences, Mountain View, CA, USA)	RT-qPCR	• Higher levels in serum EVs than in whole serum. • Higher levels in PCa patients vs. BPH patients (3.85-fold, *p* = 0.0007) and healthy controls (4.06-fold, *p* = 0.0005). • EV miRNA levels associated with PSA ≥ 10, GS ≥ 8, and T3/T4 stages. • Higher levels in LPCa vs. mPCa: AUC = 0.8694, sensitivity = 80%, specificity = 87.1%.	Li et al. (32)	
Differentially quantified EV miRNA panels depending on different comparison groups	Plasma	78 PCa (55 LPCa, 16 mPCa) vs. 28 HC	A filter concentrator with a 150-kDa molecular weight cutoff for MV extraction, Qiagen miRNeasy for MV-RNA extraction	Microarray (profiling of 742 miRNAs) RT-qPCR validation	• All 78 PCa vs. 28 HC: 12 differentially expressed miRNAs, 11 miRNA increased (miR-107, miR-130b, miR-141, miR-2110, miR-301a, miR-326, miR-331-3p, miR-432, miR-484, miR-574-3p, miRr-625), 1 decreased (miR-181a-2) • 55 nonmetastatic PCa vs. 28 HC: 10 differentially expressed miRNAs, 9 miRNA increased (miR-107, miR-141, miR-2110, miR-301a, miR-326, miR-432, miR-484, miR-574-3p, miR-625), 1 decreased (miR-181a-2) •16 mPCa vs. 55 nonmetastatic PCa: 16 differentially expressed miRNAs, 15 miRNA increased (miR-582-3p, miR-20a, miR-375, miR-200b, miR-379, miR-513a-5p, miR-577, miR-23a, miRr-1236, miR-609, miR-17, miR-619, miR-624, miR-198, miR-130b), 1 decreased (miR-572)	Bryant et al. (33)	
miR-141 ↑ miR-375 ↑	Serum	47 recurrent PCa vs. 72 nonrecurrent PCa	ExoMiR extraction kit	TaqMan RT-qPCR (miR-375 and miR-141)	• miR-141 and miR-375 were associated with recurrent (metastatic) PCa following radical prostatectomy.	Bryant, et al. (33)	
miR-107 ↑ miR-574-3p ↑	Urine (thawed cell pellets)	118 PCa (70 LPCa, 48 advanced PCa) vs. 17 HC	mirVana kit (Ambion)	RT-qPCR (miR-107, miR-574-3p, miR-375, miR-200b, miR-141)	• PCa vs. HC: miR-107 and miR-574-3p were present at significantly higher concentrations. • Both 2 miRs appeared more accurate than *PCA3* normalized to urinary PSA (AUC: miR-107 = 0.74; miR-574-3p = 0.66; *PCA3 = *0.61).	Bryant et al. (33)	
miR-1290 ↑ miR-375 ↑	Plasma	Training cohort: 23 CRPC Validation cohort: 100 CRPC	ExoQuick Exosome Precipitation Solution (System Biosciences, Mountain View, CA, USA)	Training cohort: RNA sequencing Validation cohort: RT-qPCR (miR-1290, miR-1246, miR-375)	• miRNA levels are significantly associated with poor overall survival. • Predictive performance improved *via* a combination of ADT failure time and PSA level at the time of CRPC stage with miRNA levels, with AUC increased from 0.660 to 0.730.	Huang et al. (30)	
miR-1246 ↑	Serum	Cohort 1: 8 HC vs. 44 PCa (primarily stage IV) Cohort 2: 21 BPH vs. 25 PCa (stages IIA–III)	Total EV isolation reagent (Life Technologies)	NanoString nCounter microRNA platform TaqMan RT-qPCR	• Cohort 1: EVs miR-1246 expression can significantly discriminate between HC and PCa (AUC = 0.926), compared with serum PSA with AUC = 0.869. • Cohort 1 + 2: EVs miR-1246 observed a significant inverse correlation with PCa pathologic stage, specifically upregulated in aggressive prostate cancer. • miR-1246 was downregulated in PCa clinical tissues and cell lines and was selectively released into EVs. • Overexpression of miR-1246 in a PCa cell line significantly inhibited xenograft tumor growth *in vivo* and increased apoptosis and decreased proliferation, invasiveness, and migration *in vitro*.	Bhagirath et al. (34)	
miR-19b	Urine	20 HC vs. 14 PCa	High-speed centrifugation and filtration	RT-qPCR (miR-19b, miR-25, miR-125b, miR-205)	• miR-19b distinguished PCa patients and healthy donors with 100%/93% (total urinary EVs) and 95%/79% (exosome-enriched fraction) specificity/sensitivity and a 95%-specificity and 79%-sensitivity respectively.	Bryzgunova, et al. (35)	
miR-196a-5p ↓miR-501-3p ↓	Urine	28 PCa vs. 19 HC	Centrifugation	Screening: NGS Validation: RT-qPCR	• NGS data showed 5 miRNAs were significantly decreased in healthy control vs. PCa patients: miR-196a-5p, miR-34a-5p, miR-143-3p, miR-501-3p, and miR-92a-1-5p. • Both miR-196a-5p (AUC = 0.73) and miR-501-3p levels were decreased in PCa patients (AUC = 0.69).	Rodriguez et al. (29)	
miR-2909 ↑	Urine	(90 PCa/60 BCa) vs. 10 BPH vs. 50 HC	Exiqon miRCURY™ exosome isolation kit	RT-qPCR (miR-2909, miR-615-3p)	• Urinary EV miR-2909 distinguishes PCa from BCa. • Urinary EV miR2909 correlates with the severity (GS) of PCa in all its forms. • Urinary EV miR-2909 revealed a better diagnostic potential for PCa than either PSA or miR-615-3p.	Wani et al. (36)	
miR-21 ↑ miR-375 ↑ Let-7C ↑	Urine	60 PCa vs. 10 HC	Differential centrifugation	RT-qPCR (miR-21, miR-141, miR-214, miR-375, Let-7C)	• EVs miR-21, miR-375, and Let-7C were significantly upregulated in PCa vs. HC, but no differences were found for miR-141. • A panel combining miR-21 and miR-375 is suggested to distinguish PCa patients and healthy subjects (AUC of 0.872). • Let-7C was significantly correlated with PCa clinical stage.	Foj, et al. (37)	
miR-574-3p ↑ miR-141-5p↑ miR-21-5p↑	Urine	35 PCa vs. 35 HC	Lectins, phytohemagglutinin, and concanavalin A (Sigma-Aldrich, Russia) induce agglutination of EVs	RT-qPCR	• PCa vs. HC: the levels of miR-574-3p, miR-141-5p, and miR-21-5p were significantly upregulated associated with PCa. • miR-574-3p: AUC = 0.85, miR-141-5p: AUC=0.86, miR-21-5p: AUC=0.65.	Samsonov, et al. (38)	
miR-21 ↓ miR-375 ↓ miR-204 ↑	Urine	Training cohort: 4 HC vs. 9 PCa Validation cohort: 26 HC vs. 48 PCa	Differential (ultra)centrifugation	Training cohort: NGS Validation cohort: a stemloop RT-PCR	• PCa vs. HC: NGS identified the top 10 PCa differentially expressed (Log2 fold change > 2) miRNAs in urinary EVs. Higher levels in PCa: miR-10a-5p, miR-204-5p, miR-30a-3p; lower levels in PCa: miR-375, miR-21-5p, miR-141-3p, Let-7C-5p, miR-26b-5p, miR-101-3p, Let-7b-5p. • The diagnostic performance of 3 isomiR combination of miR-21, miR-375, and miR-204 resulted in an AUC of 0.866, compared to a PSA AUC of 0.707 and the corresponding 3 mature microRNAs (miRs) AUC of 0.766.	Koppers-Lalic et al. (31)	
miR-145 ↑	Urine	60 PCa vs. 37 BPH vs. 24 HC	Hydrostatic filtration dialysis (HFD)	qRT-PCR (miR-572, miR-1290, miR-141, miR-145)	• The level of miR-145 in urine EVs was significantly increased in PCa patients compared with BPH patients. • The level of miR-145 in urine EVs was significantly increased when PCa patients with GS ≥ 8 compared with GS ≤ 7. • miR-145 in UEVs combined with serum PSA (AUC = 0.863) could differentiate PCa from BPH better than PSA alone (AUC= 0.805).	Xu et al. (39)
5 downregulated and 26 upregulated miRNAs	Urine	3 PCa (age 58, stage 4; age 57, stage 2a; and age 54; stage 2b) vs. 3 HC (ages 53, 60, and 50)	Nanowires anchored into a microfluidic substrate	Microarray (2,565 human miRNA probes)	5 downregulated: miR-15a-3p, miR-135b-5p, miR-520c-3p, miR-4783-5p, miR-7849-3p 26 upregulated miRNAs: miR-4531, miR-28-5p, miR-103a-2-5p, miR-105-5p, miR-124-3p, miR-151a-5p, miR-151b, miR-200a-5p, miR-300, miR-424-3p, miR-519c-5p, miR-551b-5p, miR-617, miR-873-3p, miR-921, miR-1288-3p, miR-3124-5p, miR-3155a, miR-3917, miR-4283, miR-4727-3p, miR-5096, miR-5187-5p, miR-6074, miR-6874-5p, miR-6892-5p	Yasui et al. (40)	
*PCA3* lncRNA ↑ *ERG* mRNA ↑	Urine	106 controls (Bx Neg) vs 89 PCa (Bx Pos)	Urine Clinical Sample Concentrator Kit (Exosome Diagnostics)	RT-qPCR computed EXO106 score (the sum of normalized *PCA3* and *ERG* RNA levels)	• EXO106 score demonstrated good clinical performance in predicting biopsy results for both any PCa (AUC = 0.715) and high-grade PCa (AUC = 0.764). • The clinical performance was improved with a combination of EXO106 and SOC (standard of care = PSA, age, race, or family history) (any PCa: AUC = 0.715; high-grade PCa: AUC = 0.803).	Donovan et al. (41)	
*PCA3* lncRNA ↑ *ERG* mRNA ↑	Urine	Training cohort: 255 patients with PSA (2 to 20 ng/ml) and biopsy outcomes Validation cohort: 519 patients with PSA (2 to 20 ng/ml) and prognostic score	Urine Clinical Sample Concentrator Kit (ExoDx Prostate IntelliScore)	Urine exosome gene expression assay (the ExoDx Prostate IntelliScore urine exosome assay): RT-qPCR	• Training cohort: gene expression assay in combination with SOC (AUC = 0.77) significantly improved performance SOC alone (AUC=0.66) for predicting high-grade PCa (HGPCa, GS ≥ 7) from low-grade PCa (GS = 6) and benign disease. • Validation cohort: compared with the Prostate Cancer Prevention Trial Risk Calculator (PCPTRC) (AUC = 0.62) and PSA alone (AUC = 0.55), Gene Expression Assay (AUC = 0.71) demonstrated improved performance. • Using a predefined cut point, 27% of biopsies would have been avoided, missing only 5% of patients with dominant pattern 4 high-risk GS7 disease.	McKiernan et al. (42)	
*TMPRSS2:ERG* mRNA ↑	Urine (urine collected prior to RP)	21 PCa: urinary EVs vs. corresponding prostatectomy tissue from the same patients 39 PCa (Bx Pos) vs. 47 controls (Bx Neg)	Filtration: a 100-k MWCO filtration concentrator (Millipore)	RT-qPCR (mRNA of *TMPRSS2:ERG*, *BIRC5*, *ERG*, *PCA3*, and *TMPRSS2*)	• Urinary EVs had a sensitivity: of 81% (13/16), specificity: of 80% (4/5), and an overall accuracy: of 81% (17/21) for non-invasive detection of *TMPRSS2:ERG* vs RP tissue. • The rate of *TMPRSS2: ERG* exoRNA detection was found to increase with age and the expression level correlated with Bx Pos status. • *TMPRSS2: ERG*: AUC = 0.744, *AR*: AUC = 0.558, *BIRC5*: AUC = 0.674, *ERG*: AUC = 0.785, *PCA3*: AUC = 0.681.	Motamedinia et al. (43)	
*PCA3* lncRNA ↑ *ERG* mRNA ↑ *KLK3* mRNA ↑	Urine (after DRE)	15 PCa (Bx Pos) vs. 14 controls (Bx Neg)	Filtration through a 100-kDa filter (Vivaspin^®^)	RT-qPCR	• The biomarker levels were highest in whole urine and significantly higher after DRE in all substrates (whole urine, cell pellet, and EVs). • In the EVs substrate of urine, no significant differences were found in *PCA3*, *ERG*, and *KLK3* between Bx Pos and Bx Neg PCa patients.	Hendriks et al. (44)	
*PCA3* lncRNA ↑ *ERG* mRNA ↑ *KLK3* mRNA ↑	Urine (after DRE)	12 HC vs. 14 GS = 6 PCa vs. 26 GS ≥ 7 PCa	Ultrafiltration	TaqMan qPCR	• EVs RNA were richer than RNA from cell pellets in urine. • PCa vs. HC: In comparison to HC, both of the GS6 and GS7+ PCa groups had significantly higher expression of *PCA3*, while only the GS7+ group had significantly higher expression of *ERG* (no difference between the 2 GS groups). • No significant difference in *KLK3* expression between any of the groups.	Pellegrini et al. (27)	
lncRNA-p21 ↑	Urine (after DRE)	30 PCa vs. 49 BPH	Urine Exosome RNA Isolation Kit (Norgen Biotek, Canada)	RT-qPCR (lncRNA-GAS5 and lncRNA-p21)	• lncRNA-p21 showed significantly higher levels in PCa vs. BPH (AUC = 0.663). • lncRNA-GAS5 showed no difference • EV lncRNA levels showed no correlation with the clinical stage (GS).	Isin et al. (45)	
SChLAP1 ↑	Plasma s	30 HC vs. 46 BPH vs. 34 PCa	Total Exosome Isolation Reagent (from plasma) (Invitrogen, Carlsbad, CA, USA)	RT-qPCR	• SChLAP1 expression was significantly higher in the PCa group than in the controls (AUC = 0.8697). • The AUC of the combination of SAP30L-AS1 and SChLAP1 was 0.9224. • AUC for SChLAP1 combined with PSA was 0.9516.	Wang et al. (46)	

↑: The expression was increased in PCa.

↓: The expression was decreased in PCa.

From a clinical perspective, 90% of the studies (19/21) have focused on the early diagnosis of PCa, which aims to distinguish PCa patients from BPH or health control. Only two studies have been designed to predict hormone resistance (30, 33). In fact, this is inconsistent with the actual needs of PCa diagnosis and treatment, such as distinguishing PCa with high metastasis risk and predicting the progression to CRPC. The main reason for taking the easy way for screening is the difficulty of collecting accurately grouped specimens in advanced PCa. One recent study focused on the differences between different PCa stages. They compared one case each in the organ-confined PC (OC) group, the extracapsular-extending PC (EC) group, and the seminal vesicle-invading PC (SI) group with healthy controls. The selected mRNAs and miRNAs with the highest change folds were different, indicating the discrimination potential related to PCa progression (47). Considering that the sample size was too small, the study was not included in **Table 2**.

From a methodological point of view, after the potential EV RNA markers were screened out through NGS or microarrays, RT-qPCR was usually adopted for further validation. The final diagnostic performance was shown in the receiver operating characteristic curve (ROC) and area under the curve (AUC). In general, the promising PCa early detection EV miRNA biomarkers include miR-141, miR-375, and miR-1246 from blood (serum or plasma) and miR-141, miR-375, Let-7C, miR-2909, miR-145, miR-21, miR-574-3p, miR-19b, miR-196a-5p, and miR-501-3p from urine. With better sampling potential than blood, urinary EVs have a broader RNA biomarker spectrum with mRNA of ERG, TMPRSS2:ERG genes (27, 41–44), and lncRNA PCA3 and p21 (27, 41, 42, 44, 45). However, up to now, no higher-level evidence has been found in studies on PCa EV RNA markers in either blood or urine samples, such as meta-analysis, which is also the premise for further clinical transformation. After all, the ultimate goal of tumor biomarker screening is not just for research, but for clinical detection.

Notably, based on data from a single study, PCa is the cancer type that is at the forefront of clinical transformation. In 2016, Exosome Diagnostics company passed FDA approval and launched the first PCa EV diagnostic product ExoDx^®^ Prostate (IntelIScore), which was certificated to have a good clinical performance (AUC = 0.77). It is also one of the only two available commercial EV kits for tumor detection until now. The other is the EV blood test kit ExoDx Lung (ALK) for detecting EML-4-ALK mutations in patients with nonsmall cell lung cancer (NSCLC). ExoDx^®^ Prostate is based on urinary EVs and provides a score by detecting the transcripts (mRNA) of PCA3 and TMPRSS2:ERG genes binding with standard of care (SOC = PSA, age, race, and family history of PCa). This method could help determine whether a prostate biopsy is needed by assessing the risk for high-grade (GS ≥ 7) and more aggressive PCa and has proven that 27% of biopsies would have been avoided (42). This study finally achieved the first completely noninvasive, nondigital rectal examination (DRE), urine-based liquid biopsy test of Exosome Diagnostics Company, which is a landmark for EV clinical diagnosis transformation.

## 4 EV DNA biomarker in PCa

At present, the research on EV nucleic acid biomarkers is mainly focused on EV RNA, while the research on EV DNA is just starting. Since 2014, some studies have gradually characterized the properties of EV DNA. First, how the DNA is extracted determines which part of the DNA on the EVs is studied. EV DNA consists of two parts, including those that adhere to the outside of the EV (external DNA) and those that are wrapped inside the EV (internal DNA). In order to better characterize the internal DNA, which is relatively impervious to external interference, EVs were pretreated with DNase and RNase to digest the external EV DNA before lysis with Triton X (48). Second, the focus is on how to characterize DNA traits. Thakur et al. demonstrated the double-stranded structure of EV DNA visually for the first time through enzyme digestion and atomic force microscopy. Gel electrophoresis further confirmed the molecular weight difference between EV DNA and genomic DNA (gDNA) in melanoma, which were mainly concentrated at 250 bp~2.5 kb and 2.5~10 kb, respectively. Despite being highly fragmented, EV DNA has been shown, without specific fragments, to be highly enriched or consumed compared with gDNA by high-throughput whole-genome sequencing (22, 49). Quantitative analysis showed that EV DNA could be detected in all tumor types, including PCa (22RV cell line), at a concentration ranging from 0 to 10 ng/µg of EV protein (22). Thirdly, the mechanism by which EVs transmit dsDNA is still controversial. It is currently considered that cells can maintain homeostasis by excreting harmful DNA through EVs (48). In addition to being a method of excretion of waste, other studies confirmed that EVs horizontally transfer genes between cells and can educate downstream cells. It has been reported that tumor EVs carry oncogene sequences such as c-Myc to transmit the genetic information of “mutation and amplification” to normal cells (50).

As PCa biomarkers, EV DNA is gradually showing some diagnostic potential, which is deployed relatively earlier and more mature than the other tumors. However, relevant studies are still relatively few, and there are still many gaps. PCa EV subpopulations carry different gDNA fragments (51) with large PCa EVs (1–10 µm) carrying most DNA (23), which indicates that the DNA molecules are selectively and cell-dependently packed into specific EV subtypes. EV DNA derived from LNCaP and PC-3 cells was sequenced to detect PCa-related mutations of MLH1, PTEN, and TP53 genes. A frameshift mutation (delAA) in codon 6 of the PTEN gene and a point mutation (C>G) in codon 215 of the TP53 gene was detected, the same as in their parental tumor cells (51). The above results showed that EV DNA has the same potential as circulating tumor DNA (ctDNA) to detect tumor-specific genetic mutations, which prompts its potential as an alternative target for gDNA from tumor tissue or cells.

## 5 Conclusion and perspectives

Based on body fluid specimens like blood, liquid biopsy can overcome tissue heterogeneity and the difficulty of sampling, which have great potential for use as noninvasive diagnostic biomarkers compared with traditional tissue biopsy. As one of the three targets of liquid biopsy, EVs have some obvious advantages. On the one hand, the collection of blood and urine to obtain EVs is easily available and minimally invasive, which helps facilitate repeated sampling and achieve dynamic and real-time monitoring of molecular changes in tumors. On the other hand, the accuracy of tumor detection could be improved by analyzing tumor-derived EVs or even selecting specific subtypes of tumor-derived EVs to increase analytical sensitivity and specificity. More recent efforts have demonstrated that tumor-derived EVs have the potential to be used as a reliable source of cancer-related diagnostic biomarkers. In particular, the nucleic acid component is stable and easy to detect and seems to be the most promising biomarker for EVs. Since EV miRNA was first reported in 2007, publications about EV RNA biomarkers have grown exponentially. Apart from miRNA, other EV nucleic acid molecules, such as oncogenic mRNA (including fusion genes and splice variant transcripts), lncRNA, and dsDNA (including cancer-driven mutation genes), also attract lots of attention. The richness of the contents, the stability of the double-layer membrane structure, and the diversity of detection methods of EVs make them have unique advantages in tumor diagnosis.

Researchers have found that EVs in biofluids contain a group of proteomic and genetic signatures of cancer, thus presenting an enormous opportunity in terms of cancer diagnosis. However, this is technically challenging due to the lack of effective and standard methods for enriching tumor-derived EVs from total EVs distinguish from EVs secreted by normal cells or specific-size EVs from total tumor-derived EVs (isolate large vesicles to extract DNA). Therefore, obtaining high purity and selected EVs is necessary before further exploration of their molecular mechanism and function. So far, many EV isolation and purification approaches have been reported, including ultracentrifugation (UC), filtration, chemical precipitation, affinity-binding beads, and microfluidics techniques. The choice of EV isolation method depends on the sample source, molecule to be detected, and downstream detection methods. UC and density gradient ultracentrifugation (DG-UC) have always been regarded as the most common approaches used to isolate total EVs. However, sequential centrifugation is time-consuming (90 min times two rounds) and instrument-demanding, and it has the problem of co-purification of non-EV-related proteins (protein aggregates and lipoproteins). In recent years, immunocapture using antibodies to target tumor-specific proteins on EV surface (e.g., PSMA or EpCAM for PCa (46)) has been optimized and has emerged as a viable choice for EV purification. In addition, the separation method of EVs should be simplified for clinical application practice.

In addition to the concerns over isolation approaches, there are further uncertainties over protocol standardization and how to define the preanalytical and analytical variables that impact outcome measures. It remains unclear how to translate omics techniques and omics information into early PCa diagnosis and prognosis forecasting. Large-scale clinical translation has not yet been carried out, and the majority of EV biomarker papers now only discuss the sensitivity, specificity, or AUC of the diagnostic model. However, it should be noted that if EVs are to be introduced into clinical laboratories as tumor markers, comprehensive verification work must be conducted to generate enough data that can define the test’s performance. The information for a mature kit should be applied, such as sample type, sampling method, anticoagulant, transportation and storage conditions, and analysis parameters including accuracy, sensitivity, specificity, linearity, lower detection limit, measurement uncertainty, etc. However, due to the need for large-scale and rigorous performance verification before clinical application, this process is time-consuming and costly. Therefore, there is still a long but promising journey between these novel EV biomarkers and their clinical translation for routine diagnostic use.

## Author contributions

YD, XH, and JYL contributed to the conception and design of the study. BZ organized the database. TD performed the statistical analysis. YD wrote the first draft of the manuscript. RL, JJL, LY, and LZ wrote sections of the manuscript. All authors contributed to the article and approved the submitted version.
